# Magnetic Alignment in Carps: Evidence from the Czech Christmas Fish Market

**DOI:** 10.1371/journal.pone.0051100

**Published:** 2012-12-05

**Authors:** Vlastimil Hart, Tomáš Kušta, Pavel Němec, Veronika Bláhová, Miloš Ježek, Petra Nováková, Sabine Begall, Jaroslav Červený, Vladimír Hanzal, Erich Pascal Malkemper, Kamil Štípek, Christiane Vole, Hynek Burda

**Affiliations:** 1 Department of Forest Protection and Wildlife Management, Faculty of Forestry and Wood Sciences, Czech University of Life Sciences, Praha, Czech Republic; 2 Department of Zoology, Faculty of Science, Charles University in Prague, Praha, Czech Republic; 3 Department of General Zoology, Faculty of Biology, University of Duisburg-Essen, Essen, Germany; University of Houston, United States of America

## Abstract

While magnetoreception in birds has been studied intensively, the literature on magnetoreception in bony fish, and particularly in non-migratory fish, is quite scarce. We examined alignment of common carps (*Cyprinus carpio*) at traditional Christmas sale in the Czech Republic. The sample comprised measurements of the directional bearings in 14,537 individual fish, distributed among 80 large circular plastic tubs, at 25 localities in the Czech Republic, during 817 sampling sessions, on seven subsequent days in December 2011. We found that carps displayed a statistically highly significant spontaneous preference to align their bodies along the North-South axis. In the absence of any other common orientation cues which could explain this directional preference, we attribute the alignment of the fish to the geomagnetic field lines. It is apparent that the display of magnetic alignment is a simple experimental paradigm of great heuristic potential.

## Introduction

While magnetoreception in birds has been studied intensively, the literature on magnetoreception in bony fish is quite scarce. The first evidence for magnetosensation was based on the finding of a spontaneous preference for cardinal compass directions in the resting European eel, *Anguilla anguilla* [1], [2], the goldfish, *Carassius auratus* [3], and the rainbow trout, *Oncorhynchus mykiss* [4]. The European eel preferred south-south-western oriented tubes placed within a pond over West-North-Western oriented tubes [5]. Young sockeye salmons (*Oncorhynchus nerka*) placed in a circular arena preferred the direction corresponding to their natural migratory direction and changed this directional preference according to an experimental shift of the magnetic field polarity, yet they did not react to changes of inclination, a clear indication of polarity-based compass [6]–[8]. Behavioral sensitivity to magnetic field was found also in the larvae and fry of the common trout, *Salmo trutta* [9]. On the other hand, there was no observable effect on the horizontal and vertical movements of the chum salmon (*O. keta*) when the magnetic field was modified [10]). Spontaneous bimodal magnetic preference has recently been demonstrated in the zebrafish (*Danio rerio*) [11]. The other line of evidence for magnetosensation is based on conditioning experiments. The yellowfin tuna (*Thunnus albacares*) [12]), the rainbow trout [13], [14], the zebrafish (*Danio rerio*) and the Mozambique Tilapia (*Tilapia zillii*) [15]) could be trained to discriminate between two magnetic fields of different intensities. In contrast to that, conditioning to magnetic stimuli failed in the atlantic salmon (*Salmo salar*, [16]), the goldfish [17], and the land-locked sockeye salmon [18]. Magnetic field responsiveness in the Japanese eel (*Anguilla japonica*), the darkbanded rockfish (*Sebastes inermis*), and the rainbow trout was demonstrated by a heartbeat conditioning test [19]–[23]. Indirect evidence for magnetoreception in fish comes from the observations that there were significantly more fish (European perch, *Perca fluviatilis*; common roach, *Rutillus ruttilus*; common rudd, *Scardinius erythrophthalmus*; common bleak, *Alburnus alburnus*) trapped in fyke nets equipped with ferrite magnets mounted at the entrances to the fyke nets than in control nets [24]. The other line of indirect evidence for magnetoreception in fish comes from finding putative magnetoreceptor cells in the olfactory sensory epithelium containing conspicuous iron-rich crystalline inclusions with magnetic properties consistent with single domain magnetite [25]–[28]. Taken together, the research on magnetoreception in fish focused particularly on migratory fish species, especially on salmonids and eels. The behavioral evidence concerning cyprinid species remains limited.

**Figure 1:**
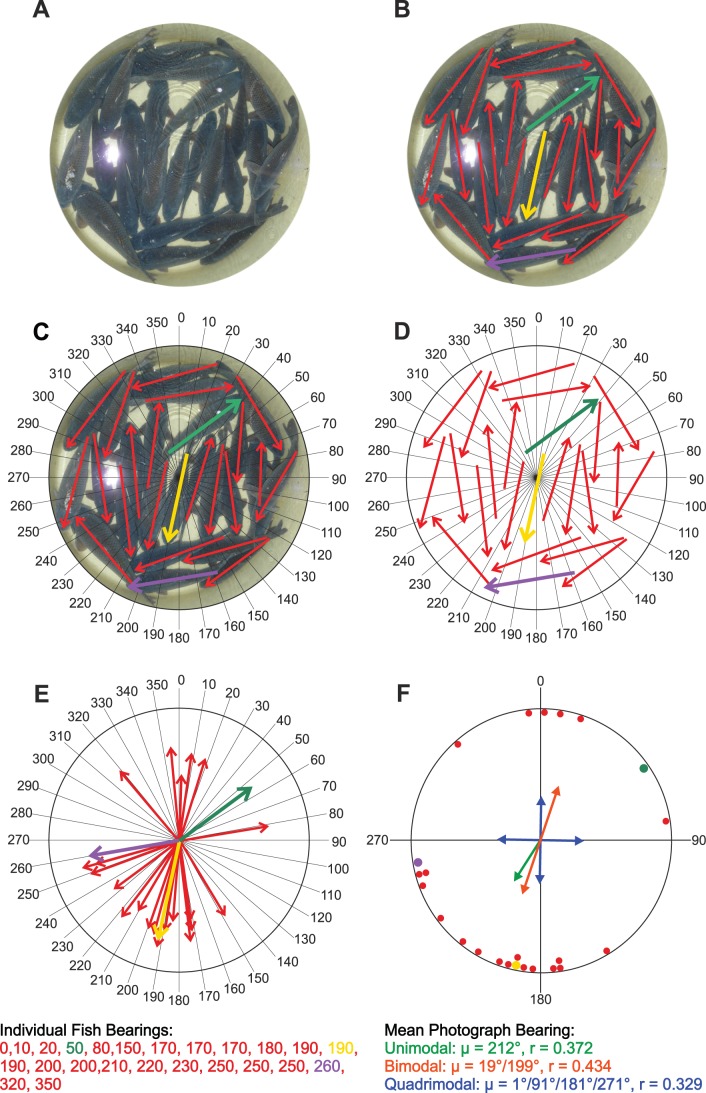
Graphic demonstration of the measuring of carps’ bearings. (**A, B**) Arrows were drawn along the median axes of all the fish visible in the photograph (the arrow axial course was marked unambiguously by the long dorsal fin, its direction by the head position). (**C, D**) The underlying photograph was removed and replaced by a compass rosette divided radially into 36 ten-degree segments. (**E**) Each arrow was moved to the center of the rosette and its azimuthal direction was determined by the nearest 10° mark. (**F**) Mean unimodal, bimodal and quadrimodal vectors were calculated for the photograph.

The common carp (*Cyprinus carpio*), one of the economically most important freshwater cyprinid species, used to be a favorite edible fish in the Roman Empire. It was domesticated in China and Europe and aquacultured for centuries. In East Asia the carp became popular as koi, while outside Europe and Asia it is known mostly as an invasive nuisance fish species (especially in Australia). It used to be considered a non-migratory, sedentary fish, yet recent studies have shown that under natural, unrestrained conditions in rivers, carps can undertake long movements of up to several hundred kilometers [29]–[31].

The carp is one of the traditional Christmas Eve meals in Central and East Europe. In the Czech Republic (population of about ten million people), about 14 thousand tons of carps, (i.e., about 5–6 million individual fish) are sold yearly during Christmas time. The vendors traditionally keep the fish for sale in large plastic (formerly wooden) circular tubs in the streets of cities and in villages during the last week before Christmas Eve, and many buyers take the fish home alive and keep them till 24th December, so that the meat is fresh; or alternatively they buy and release them, also as a part of Christmas customs, back in ponds and rivers. We exploited this unique tradition as an extraordinary large-scale experiment to test the hypothesis of magnetoreception in cyprinid fish.

**Figure 2:**
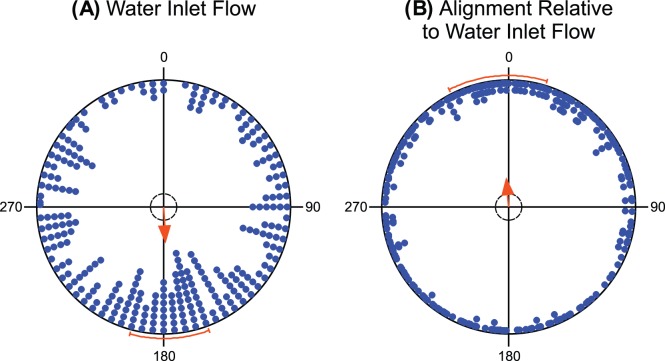
Fish alignment relative to water inlet flow. (**A**) Distribution of the water inlet flow directions. (**B**) The photograph mean bearings plotted relative to the direction of the water inlet flow (standardized to 0°). Arrows indicate the mean vector for the distribution, the length of the mean vector provides a measure of the degree of clustering in the distribution. The inner dashed circles mark the 5% significance border of the Rayleigh test; the arrows exceeding these circles indicate significant directional orientation. The bars outside of the circles delimit 95% confidence interval for the mean bearings.

## Materials and Methods

### Animals

Common carps (*Cyprinus carpio*) were kept outside (i.e., at ambient temperatures) in circular tubs made of hard plastic. The tubs were usually white, blue or pale brown, 60 cm tall (deep) and with an inner diameter of 120 cm. To supply a sufficient amount of oxygen and to prevent freezing of water, a continuous inflow of fresh water was provided by a hosepipe placed about 15 cm above the water level. No food was provided.

**Figure 3:**
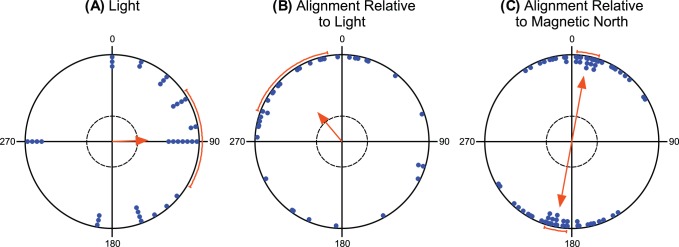
No fish alignment relative to light. (**A**) Distribution of the nearest or the strongest light source positions. (**B**) The tub mean bearings plotted relative to the position of the light source (light positions standardized to 0°). See caption to Fig. 2 for explanation. (**C**) The tub mean bearings plotted relative to the magnetic North. Each pair of dots (located on the opposite sites within the unit circle) represents the direction of the bimodal mean tub vector (see Methods). The double-headed arrow indicates the grant mean axial vector calculated over all tubs; the length of the grant mean vector provides a measure of the degree of clustering in the distribution of the tub mean vectors.

**Figure 4:**
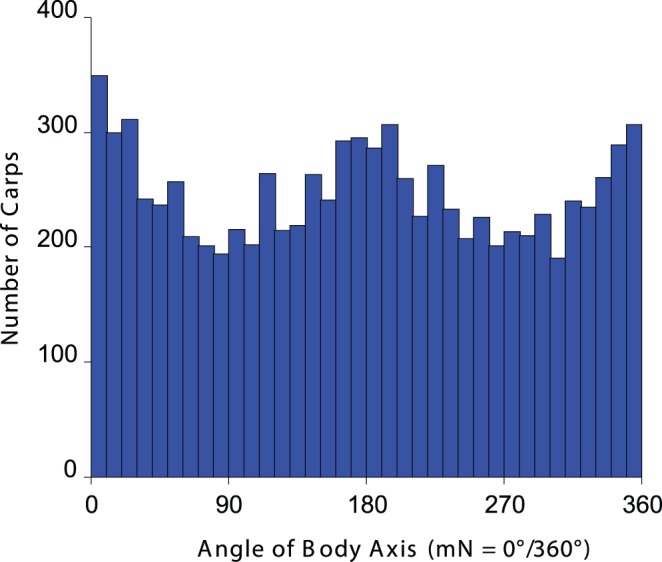
Histogram showing the distribution of individual fish directional bearings in circular tubs. Note preferential alignment in the North-Southern direction.

**Figure 5:**
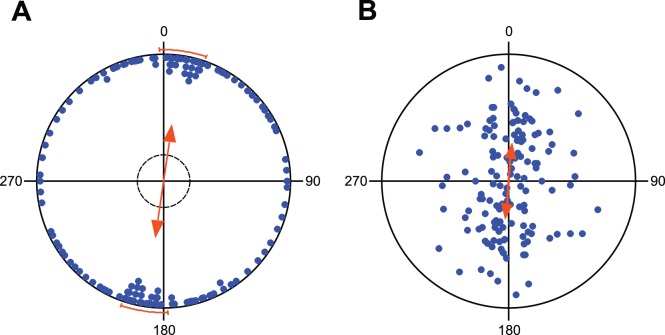
Circular diagrams of tub mean vector distributions demonstrating the North-Southern alignment in carps. (**A**) Raw data plot. Each pair of dots (located on the opposite sites within the unit circle) represents the direction of the bimodal mean tub vector. The double-headed arrow indicates the grant mean axial vector calculated over all tubs; the length of the grant mean vector provides a measure of the degree of clustering in the distribution of the tub mean vectors. The inner dashed circle marks the 5% significance border of the Rayleigh test; the bars outside of the circle delimit 95% confidence interval for the grand mean bearing. (**B**) Scatter plot summarizing statistics weighted by the length of the mean vectors for individual tubs. The position of each pair of dots within the circle represents both the direction and the length of the bimodal mean vector for one tub. The double-headed arrow indicates the weighted grant mean axial vector calculated over all tubs.

### Ethics Statement

Our study was based on making photographs of the containers (tubs) with the fish legally sold by vendors on the fish market. The sale of this fish in the Czech Republic is approved and strictly controlled by the State Veterinary Service of the Czech Republic, the Ministry of Agriculture, and the State Agricultural and Food (Processing) Inspection of the Czech Republic. Taking photographs of the fish did not affect their behavior in any way and was a fully non-invasive method of the study. Therefore no special permits were necessary for our observations. Any accompanying manipulation of the fish such as temporary switching off the water inlet did in no way exceed routine manipulation during keeping and selling the fish.

### Photic and Magnetic Conditions

*S*ales stalls were typically located in busy streets/squares or in front of stores/supermarkets. Consequently, standardization of light and magnetic conditions was not possible. Except for a few localities, light pollution, a by-product of street lights, was omnipresent. Street lighting created a clear light gradient at nine localities studied. A significant amount of electromagnetic pollution, which may potentially disrupt magnetosensation (see e.g. [32], [33]), is also to be expected at the localities studied. However, we only measured parameters of the static magnetic field using a GeoMag three-axis digital magnetometer fitted with a Honeywell HMR2300 probe sensor (Edis vvd, Kosice, Slovakia). At all localities but one, the mean total intensity (48±2 µT), and inclination (66°±3°) of the ambient magnetic field were comparable to the properties of the local geomagnetic field. However, it has to be noted that irregular oscillations of the field intensity were recorded. These changes were usually small in comparison to the total intensity of the ambient field but ranged up to 1.4 µT in some cases.

### Sampling

Eight of us (V. Hart, T.K., M.J., P. Nov., K.Š., J.Č., V. Han., and H.B.) have collected 817 digital photographs of 80 tubs with carps at 25 localities in Prague and its environs, and in several cities in Northwest and South Bohemia between 18th and 24th December 2011. On average, there were three tubs at each locality (sales booth) and each locality was revisited 5 times during the following seven days. Photographs were taken when the fish had been left undisturbed for at least 3 minutes. Consequently, most recordings took place during late evening or at night, after closing time. It should be pointed out that carps were habituated to the street tubs and did not visibly react upon the proximity and/or movement of observers. Photographs of the tubs were taken from above, and the azimuthal direction was marked on the rim of the tub so that the position of magnetic North could be recognized in each photograph. If there was a water inlet turned on, we recorded its position, took a photograph, shut it off, removed the hosepipe and stirred the fish using a dip net. Then, we waited till the fish calmed down (after about three minutes), and took a second picture. We also recorded the air and water temperature, position of street lighting, and other potentially disturbing factors.

### Data Analysis and Statistics

Before analysis, all photographs were cropped using the ellipse mask tool within the Corel Photo-Paint software (Corel Corporation, Ottawa, Ontario, Canada) to remove the rim of the tubs, azimuthal marks, and all other unwanted outer parts of the images. The resulting pictures were coded, and subsets (about 30% of the photographs) were rotated clockwise by 90°, 180°, and 270°, respectively. This data set was analyzed blindly by five members of our team (V.B., S.B., E.P.M., C.V., and one technician) that did not participate in data collection and did not know the orientation of the tubs. They measured the directional bearings (i.e., the head directions) of all carps that could be recognized in the photographs. In total, 14,537 individual fish bearings were measured (5,644 and 8,893 bearings in the tubs with and without water inlet flow, respectively). On average, ∼18 fish per photograph could be measured. The measurement procedure has been described previously [33], [34]. Briefly, the coded photographs were imported to Microsoft PowerPoint and analyzed in a three-step procedure (see Fig. 1 for a graphic demonstration). Firstly, arrows were drawn along the median axes of all the fish visible in the photograph (the arrow axial course was marked unambiguously by the long dorsal fin, its direction by the head position). Secondly, the underlying photograph was removed and replaced by a compass rosette divided radially into 36 ten-degree segments. Thirdly, each arrow was moved to the center of the rosette and its azimuthal direction was determined by the nearest 10° mark. Subsequently, the topographic bearings were recalculated to true magnetic bearings by the researcher (P. Něm.) who had rotated the photographs before the measurement.

Fish within a tub do not behave independently of each other. Moreover, since we cannot guarantee that all the fish were sold and replaced between two subsequent recordings of the same tub, it is likely that our data are partly based on repeated measurements of the same individuals. Therefore, we used second order statistics to obtain statistically independent data. First, we calculated one mean vector bearing for each photograph and subsequently the weighted (by the length of the vectors for individual photographs) mean vector for each tub. Tubs for which we collected less than three recordings, were excluded from further analyses. For the analysis of a possible effect of water current on fish orientation, we used mean vectors for photographs. Because the position of the water inlet changed quite considerably between recordings, the calculation of mean bearings for the tubs would likely obscure any relationship between the direction of the water flow and the orientation of fish. Given that the water current may potentially influence body orientation of carps, all statistical analyses were done separately for the tubs with and without water inlet flow.

Magnetic alignment might potentially be unimodal, but typically leads to bimodal or quadrimodal orientation coinciding with the magnetic cardinal directions (for review, see [35], [36]). Therefore, we performed all analyses separately for each of the three possible distributions. Thus, we calculated mean vector for each photograph in three ways: (i) from original sectors to test unimodal distribution, (ii) for test of bimodality from sectors calculated by doubling the angles method and (iii) finally, for test of quadrimodality from sectors calculated by double-doubling the angles [37]. To obtain statistically independent data, we have calculated one mean vector for each tub (see above). The Rayleigh test was used to determine whether the clustering of the mean tub bearings was greater than that expected by chance. For weighted statistics taking a vector length into account, the Moore’s modified Rayleigh test and Hotelling’s test were used to assess significant deviations from a random distribution of mean tub vectors. In cases of bimodal and quadrimodal distributions, the above mentioned tests were applied to transformed mean bearings [37]. The Mardia-Watson-Wheeler test was used to determine whether two distributions were identical, circular-circular correlations was used to analyze the effects of water flow and light gradient on carps’ body orientation [37]. Analyses involving doubling the angles and double-doubling the angles techniques were done using custom-made software, all other tests were calculated with Oriana 4.01 (Kovach Computing).

## Results

### Effect of Water Inlet Flow

*Water inlets* may potentially create complex water flow patterns in the tubs (cf. [38]). Nevertheless, the orientation of these flow patterns is, at least partly, determined by the direction of the water inlet flow. Therefore, we compared the directions of the water inlet flow with the carps’ mean angular bearings to assess whether water flow affects body orientation of the carps. Indeed, there was a weak but significant correlation between these two variables (circular-circular correlation coefficient r = 0.045, p<0.05). When the mean bearings were plotted relative to the inlet flow direction, the bearings were unimodally oriented (µ = 355° ±22° (mean vector orientation angle, 95% confidence interval), r = 0.217 (mean vector length), N = 272, p = 2.84×10^−6^; Fig. 2 B). Since the 95% confidence interval for the grand mean bearing included the inlet flow direction, it can be concluded that the carps tend to orient parallel to the direction of the water current. Because the directions of the water inlet flow were significantly biased towards south (µ = 177° ±18°, r = 0.27, N = 272, p = 2.52×10^−9^; Fig. 2A), it cannot be unambiguously discerned whether a prospective North-South alignment represents a response to water current (i.e., rheo-alignment) or magnetic alignment. Therefore, we excluded the tubs with the water inlet flow turned on from further analyses.

### No Effect of Light

Although the data were collected during winter evenings/nights (i.e., after sunset), there were street lights in the vicinity of 35 tubs at 9 localities studied. To test whether carps used a light gradient as an orientation cue, we compared the directions towards the nearest light sources with the mean axial bearings. There was no correlation between these two variables (circular-circular correlation coefficient r = 9.64×10^−4^, p>0.05). While light came predominantly from the east (µ = 88° ±33°, r = 0.395, N = 35, p = 0.004; Fig. 3A), carps aligned their bodies roughly along the North-South axis (µ = 11°/191° ±7°, r = 0.75, N = 35, p = 2.41×10^−9^; Fig. 3C). The distribution of light positions differed significantly from the distribution of mean carp axial bearings (Mardia-Watson-Wheeler test: W = 13.12, P = 0.001). Although the carps were significantly unimodally oriented when the mean bearings were plotted relative to the position of the light source (µ = 320° ±30°, r = 0.422, N = 35, p = 0.002; Fig. 3B), it seems highly unlikely that they would align themselves 40° counterclockwise to the light gradient. Taken together, the light cues can hardly explain the observed North-South alignment, so we conclude that the light gradient did not affect the body orientation of the carps.

### Body Orientation of Carps

In the tubs without water inlet, carps aligned their bodies along the North-South axis. Figure 4 shows the body orientation of individual fish. The ratio of the number of fish in the 45° sectors around the North-South axis to the number of fish in the 45° sectors around the east-west axis equals 1.44. A statistical analysis based on independent mean bearings calculated for individual tubs confirmed a significant bimodal distribution of mean bearings (µ = 8°/188° ±10°, r = 0.437, N = 69, p = 1.85×10^−6^; weighted statistics: WMV = 6°/186°, r = 0.228, N = 69, p<0.001; Hotelling’s test: F = 49.39, p<10^−12^; Fig. 5). The 95% confidence interval for the grand mean bearing included the North-South axis.

Neither unimodal (µ = 29°, r = 0.029, N = 69, p = 0.93) nor quadrimodal (µ = 6°/96°/186°/276°, r = 0.183, N = 69, p = 0.10) distribution of the mean tub bearings were significant.

## Discussion

The observations performed in this study show that carps preferentially align their bodies along North-South axis. To the best of our knowledge, this is the first study of spontaneous directional preference in any fish species, conducted on such an extensive sample distributed in time and space. Given that our analyses were restricted to the tubs without the water inlet flow and because of the temporal and spatial distribution of independently observed fish tubs, we can exclude any common orientation cues (such as wind, temperature, noise and vibrations, light, water flow, and position and movement of people outside the tub) but the geomagnetic field. We therefore suggest that carps used the magnetic field azimuth as the primary orientation cue and interpret the observed phenomenon as a case of magnetic alignment.

The magnetic alignment and alignment-like fixed direction responses, representing a spontaneous, non-goal-directed orientation of animals with respect to the magnetic field lines, have been described in a handful of species representing, however, diverse animal taxa such as insects [39]–[41], fish [1], [3], amphibians [42]–[43]), birds [44], and mammals [33]–[35], [45]–[46]. This widespread phenomenon requires explanation. In spite of the fact that the role of the magnetic alignment has been discussed in all the above cited papers, its adaptive significance remains elusive and unstudied. Biological function of the magnetic alignment may be taxon-specific and constitutes a challenge for the future research [35]. This challenge is further increased by the present finding of spontaneous magnetic alignment in a common freshwater fish.

Spontaneous directional preference for a certain magnetic direction may facilitate building a group (school, flock, herd); moving in a given direction and maintaining the same direction, and may be important for synchronized locomotion, coordinated escape and avoiding collisions [33]–[35], [47]). We speculate that magnetic alignment may help to synchronize the direction of movement of individuals in groups, and it may also be a manifestation of the magnetic compass orientation or even navigation. With respect to the study of magnetoreception in fish, it is apparent that the display of magnetic alignment is a simple experimental paradigm of great heuristic potential.
